# Human Exposure to Metals and Potential Human Health Risk in a Volcanic Environment in Italy

**DOI:** 10.3390/toxics13121080

**Published:** 2025-12-15

**Authors:** Giovanni Forte, Venerando Rapisarda, Flavia Ruggieri, Beatrice Battistini, Lisa Bauleo, Veronica Filetti, Elena Grignani, Piero Lovreglio, Serena Matera, Paola Senia, Francesca Vella, Ermanno Vitale, Beatrice Bocca, Ivo Iavicoli

**Affiliations:** 1 Department of Environment and Health, Italian National Institute of Health, 00161 Rome, Italy; 2 Department of Clinical and Experimental Medicine, University of Catania, 95124 Catania, Italy; 3 Environmental Research Center, Istituti Clinici Scientifici Maugeri IRCCS, 27100 Pavia, Italy; 4 Interdisciplinary Department of Medicine, University of Bari, 70124 Bari, Italy; 5 Department of Medicine and Surgery, University of Enna “Kore”, 94100 Enna, Italy; 6 Department of Healthcare Surveillance and Bioethics, Catholic University of Sacred Heart, 00168 Rome, Italy; 7 Fondazione Policlinico Universitario A. Gemelli IRCCS, 00168 Rome, Italy

**Keywords:** volcano, toxic metals, urine, reference values, health risk assessment

## Abstract

Mt. Etna is the highest and most active stratovolcano in Europe, located in Catania (Sicily, Italy). Its persistent degassing, frequent explosions, and lava flows release large amounts of ash and gases into the atmosphere. This study aimed to assess whether chronic exposure to local volcanic emissions leads to an increased internal dose of trace elements (As, Ba, Be, Bi, Cd, Co, Cr, Cu, Hg, Li, Mn, Mo, Ni, Pb, Sb, Se, Sn, Sr, Tl, U, V, W, Zn) in Catania adult residents. To this end, urine samples were collected from 167 individuals residing in Catania and compared with 193 residents of other Sicilian areas located farther from the volcano. Results revealed significantly higher urinary concentrations of As, Hg, Mn, Pb, and Tl in the exposed group, suggesting volcanic activity as a relevant source of exposure. The levels of the other elements were instead affected by other factors such as lifestyle habits and the consumption of specific foods and beverages. The urinary concentrations of trace elements were consistent with reference values reported in other European studies, and the levels remained well within the health-based guidance values. There is evidence of an increased internal dose of a few elements in the Sicilian population exposed to volcano activity, but the observed increases are unlikely to pose a significant health risk.

## 1. Introduction

Volcanic eruptions can pose significant risks to nearby communities. The hazards include lava flows, tephra fallout, mudflows, and toxic gases, which can have devastating impacts on both the environment and human life [1]. Mt. Etna is the tallest (over 3000 m) and largest active stratovolcano in Europe. It is located on the eastern side of Sicily (Italy), covering a vast area of *ca*. 1200 km^2^ and it is placed at about 25 km northwest of Catania, a city with a population of over 300,000. The volcanic activity of Mt. Etna started around 600,000 years ago due to the collision between the African and the Eurasian plates. Mt. Etna is known for its continuous degassing, frequent explosions, and lava flows from its summit craters, which produce significant amounts of ash, gas, and lapilli [2]. The volcano is responsible for emitting about 16% of global volcanic heavy metals (Cu, Zn, Cd, Sn, Tl, Pb, Bi) and 19% of alkali metals (Na, Rb, K, Cs) during eruptions; even during periods of quiescence, it continues to emit around 2% and 4% of these elements, respectively [3]. Volcanic ash from Mt. Etna contains micro-crystals rich in trace elements [4]. When it settles and mixes with rainwater, these elements dissolve and enrich the subsoil, either entering aquifers or fertilizing surrounding soils. This natural process, observed during other major volcanic eruptions, enhances soil fertility and contributes to the improved quality of agricultural products [5]. Additionally, Mount Etna’s groundwater, rich in beneficial minerals, supports biological functions and is widely used for drinking, agriculture, and industry [6]. While Mt. Etna’s volcanic activity enriches the soil and water with essential nutrients, it also introduces toxic elements like Pb, Cd, Ni, and Hg [6,7]. Chronic exposure to these elements can mainly occur through ingestion and inhalation, leading to their presence in the body. Barsotti et al. [8] conducted a quantitative hazard assessment using numerical modelling to evaluate the potential human health impacts of volcanic tephra fallout from Mt. Etna, while Fano et al. [9] linked the chronic ash exposure to an increased risk of various diseases among people living on the southeastern flank of Mt. Etna, specifically in the city of Catania. Several studies reported a potential link between Mt. Etna ash exposure and various neurodegenerative diseases, including multiple sclerosis [10,11,12], amyotrophic lateral sclerosis [13], thyroid cancer [14,15,16], and cardiovascular diseases [17]. During ashfall events, eye and airway irritation, coughing, and exacerbation of chronic respiratory disorders are common issues [17]. In subjects exposed to the ash, a correlation between the frequency of Etnean ash events and the production of total immunoglobulin E (IgE) antibodies was assessed, suggesting that volcanic ash may alter the upper respiratory tract and promote allergopathies [18]. A key concern is the long-term health risk posed by volcanic ash, particularly due to its remobilization by wind or human activities such as vehicular traffic and construction. These processes can lead to inhalation hazards and the contamination of food and beverages [19,20]. Studies on children and adolescents chronically exposed to volcanic activity around Mt. Etna revealed higher levels of trace elements in scalp hair compared to individuals residing in other regions of Sicily [19,20], while in adults exposed to the 2001 Etna eruption the higher levels of trace elements in bronchoalveolar lavage (BAL) were linked to the ability of trace elements to promote the crystallization of phosphate microstructures within pulmonary tissue [21]. The Human Biomonitoring (HBM) campaign is considered one of the most effective approaches for evaluating population exposure to various chemicals. HBM measures biomarkers in human biological fluids or tissues, providing a direct assessment of the internal dose of chemicals and a more accurate picture of what people are truly exposed to [22,23,24]. Urine collection is a simple, non-invasive procedure that carries minimal risk, and monitoring metal concentrations in urine is essential for assessing volcanic exposure and informing strategies to mitigate potential health risks. The present study aims to evaluate the concentration of 23 trace elements in urine samples of adult subjects exposed to Mt. Etna’s emissions (Catania city) and control adult subjects living in other areas of Sicily located farther from the volcano. Variables such as age, residence, gender, BMI, lifestyle, food, and beverages were also considered to ascertain whether elements exposure is primarily due to volcanic emissions or influenced by other sources. By accounting for these variables, the study intends to better isolate the impact of volcanic emissions on trace element exposure and to understand the other environmental, dietary, and lifestyle factors at play. Moreover, this study also presents reference values (RVs) for the Sicilian population and uses them for the comparison with health-based guidance values (HBGVs) to estimate the health risk in terms of Hazard Index (HI) and Risk Quotients (RQs) to individual metals and mixtures of metals.

## 2. Materials and Methods

### 2.1. Sampling Areas

**Figure 1:**
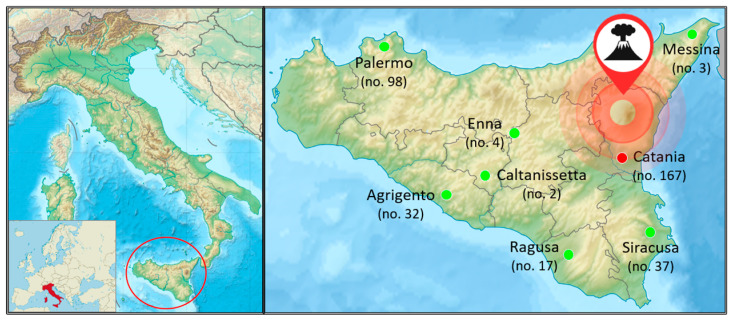
Sampling areas including Catania province as volcanic zone (<35 km from the top of Etna) and other provinces as controls (https://upload.wikimedia.org/wikipedia/commons/7/79/Italy_relief_location_map.jpg; https://upload.wikimedia.org/wikipedia/commons/3/36/Relief_map_of_Italy_Sicily_crop.svg. Accessed on 15 December 2025).

The sampling areas (Figure 1) included the following: (i) Catania; and (ii) control cities (Agrigento, Caltanissetta, Enna, Messina, Palermo, Ragusa, and Siracusa). Catania is directly affected by volcanic emissions and is considered the exposed area. Catania, with about 315,000 inhabitants, is in southeastern Sicily, facing the Ionian Sea and close to Mt. Etna. The terrain is flat to the south and southeast, and mountainous to the north due to Mt. Etna. The urban area features marine clay and sandy deposits from the Lower-Middle Pleistocene, layered with alluvial fan deposits from the Middle Pleistocene, terraced alluvial deposits from the Middle-Upper Pleistocene, and lava flows from Mt. Etna’s lateral eruptive systems from the Upper Pleistocene–Holocene [25]. Prevailing north/north-west winds at Mt. Etna push volcanic plumes east towards Catania, affecting the city and sea [26]. Control cities (north, south, west) avoid the direct plume, and their distance from Catania ranged between *ca*. 60 km (Siracusa) and *ca*. 200 km (Palermo). Cities such as Messina, Palermo, and Siracusa are situated at sea level, whilst the others are up to *ca*. 1000 m (e.g., Enna) above sea level. Finally, the geo-lithological history of the control area (sedimentary or calcareous rocks) is greatly different from the exposed area of volcanic origin [19].

### 2.2. Subjects

The study included 167 subjects residing in the province of Catania and exposed to Etna’s volcanic activity (mean age 40.4 ± 12.5 years; 66 females and 101 males) and 193 control subjects living in areas of Sicily located far from the volcano (mean age 40.1 ± 12.5 years; 49 females and 144 males). Participation in the study was voluntary.

Specific inclusion and exclusion criteria were applied for participant selection. Inclusion criteria required individuals to be between 18 and 65 years, who were healthy, followed a mixed diet, and had been residing in the same area for more than 15 years. The exclusion criteria included occupational exposure to metals, current smoking, presence of metallic prosthesis, piercings, tattoos, chronic diseases, intake of nutritional supplements or drug medications, and special diets (such as vegan or vegetarian) to minimize confounding factors that could influence urinary element levels. At the time of delivery of the urine samples, carried out at the University of Catania between 1 April 2020 and 31 March 2021, all subjects completed a detailed questionnaire regarding personal characteristics, lifestyles, diet, type of water consumed, intake of locally produced foods, and exposure to traffic at home and at the workplace. The characteristics of the subjects are reported in Table S1 (Supplementary Materials).

All questionnaire variables were checked for completeness and internal consistency before the statistical analysis. Missing data never exceeded 5% for any variable. Given the low rate and the absence of systematic patterns, missing values were handled through complete-case analysis. Dietary and lifestyle variables were categorized a priori according to frequency of intake (e.g., ≤1 time/week, 2–3 times/week, >3 times/week) or exposure (yes/no). All variables included in the models were coded as categorical dummy variables.

All subjects gave written informed consent before procedures. The study protocol was approved by the Institutional Ethical Committee of the University of Catania (protocol number 19/2020/PO, approval date 24 March 2020) and conducted according to the Declaration of Helsinki.

### 2.3. Sample Preparation

First-morning urine samples were collected in high-density polyethylene bottles (Kartell, Milan, Italy) that had been decontaminated with a 5% ultrapure nitric acid (HNO_3_) solution (VWR, Leuven, Belgium) and stored at −20 °C until analysis. After shaking, 1 mL of urine was sub-sampled from each container and added with 0.1 mL of ultrapure HNO_3_ and 3.9 mL of ultrapure deionized water (Micro Pure UV, Thermo Scientific Barnstead, Langenselbold, Germany). Quality control measures were checked against Certified Reference Materials, namely the Seronorm lyophilized human urine level 1 and level 2 (Sero AS, Billingstadt, Norway). These quality control samples were analyzed concurrently with test samples at a frequency of one per 20 samples during the sequence to monitor daily accuracy and precision [27]. Accuracy ranged between 93 and 109% at level 1 and between 94 and 101% at level 2 for all the analyzed elements. Precision on replicated measurements of CRMs was better than 8% and 4% at level 1 and level 2 for all the analyzed elements. To account for variations in urine concentration, the element data were normalized using urinary creatinine values. These values were determined through the colorimetric method (Jaffé Method, Cobas Pro, Roche Diagnostics, Mannheim, Germany) based on the reaction between creatinine and alkaline picrate, which produces a coloured compound. The amount of colour formed is directly proportional to the concentration of creatinine in the sample.

### 2.4. Determination of Elements

The Thermo Scientific iCAP Q inductively coupled plasma mass spectrometry (iCAP Q ICP-MS, Bremen, Germany) was used to determine the following elements: 75As, 138Ba, 9Be, 209Bi, 114Cd, 59Co, 52Cr, 63Cu, 202Hg, 7Li, 55Mn, 100Mo, 60Ni, 208Pb, 123Sb, 82Se, 120Sn, 88Sr, 205Tl, 238U, 51V, 184W, and 64Zn. To efficiently reduce polyatomic interferences on the analytical masses, the He pressurized QCell in Kinetic Energy Discrimination (KED) mode was employed. The addition calibration method and the use of 103Rh as an internal standard (1 ng/mL in the analytical solutions) served to mitigate matrix effects and to correct possible instrumental drifts. Limits of detection (LoDs) were calculated as 3 times the standard deviation of 10 replicates of a pooled urine sample. The LoDs resulted in the following (in µg/L): 75As, 0.92; 138Ba, 0.06; 9Be, 0.04; 209Bi, 0.11; 114Cd, 0.02; 59Co, 0.03; 52Cr, 0.05; 63Cu, 1.40; 202Hg, 0.40; 7Li, 1.87; 55Mn, 0.03; 100Mo, 1.44; 60Ni, 0.12; 208Pb, 0.13; 123Sb, 0.015; 82Se, 2.00; 120Sn, 0.04; 88Sr, 7.16; 205Tl, 0.07; 238U, 0.01; 51V, 0.05; 184W, 0.03; 64Zn, 17.1. Performances of the ICP-MS method are reported in Table S2 (Supplementary Materials). A concentration equal to LoD/2 was assigned to elements below their respective LoDs.

### 2.5. Statistics

The Kolmogorov–Smirnov test was performed to assess the data distribution. As the dataset was found to be non-normally distributed, creatinine-normalized element concentrations were reported as median (P50) and 5th–95th percentiles (P5–P95), along with a description of the sample size and number of samples below the LoDs. Differences between creatinine-normalized element concentrations in exposed and control subjects were tested by the Mann–Whitney non-parametric test.

For each element, multivariate regression models were built using log-transformed creatinine-normalized urinary concentrations as dependent variables. Gender, age, and exposure group (exposed vs. control) were forced into all models as a priori confounders (lockterm option). Additional covariates were selected using a stepwise forward selection procedure with a probability of entry set at *p* < 0.20. The predictors included BMI, type of drinking water, fish, bread, pasta, etc. (Table S3). Bootstrap resampling based on 100 replications internally validated the stability of the variable selection. Variables consistently selected across bootstrap samples were retained in the final models. Given the categorical structure of the predictors and the stepwise procedure, a low degree of multicollinearity among covariates was expected. Nevertheless, collinearity was formally assessed using the variance inflation factor (VIF). All VIF values were consistently below 2.5, indicating the absence of relevant multicollinearity.

Associations were expressed as geometric mean ratios (GMRs), obtained by exponentiating the regression coefficients, with 95% confidence intervals.

The Linear Discriminant Analysis (LDA) classification model was made using the prior probability based on the group size to assign each individual to a class according to type of population. The IBM SPSS Statistics version 28 (IBM Corp., Armonk, NY, USA) and STATA version 16 (Stata Corp, College Station, TX, USA) software programmes were used for the statistical analyses.

### 2.6. Risk Assessment

The human risk was determined as the individual exposure to each single metal or to their combination as a mixture. The risk for an individual metal was identified by the RQ, calculated as the ratio between the P50 and P95 concentration of each metal ($C_{i}$) and the respective HBM HBGV_i_:

$${RQ}_{i}=\frac{C_{i}}{{HBM\;HBGV}_{i}}$$

In the case of a mixture of metals, the HI, calculated as the sum of each RQ_i_, evaluated the human risk:

$$HI=\sum_{i=1}^{n}{RQ}_{i}$$

If the RQ and HI were <1, the single metal or the mixture of metals did not pose a risk [28].

## 3. Results

All samples met the creatinine concentration (P50, 0.75 g/L; range, 0.30–2.01 g/L) criteria established by the WHO guidelines (0.30–3.0 g/L). Table 1 reports the element concentrations (µg/g creatinine) detected in urine of the total, exposed, and control groups. According to the Mann–Whitney test, 8 elements out of 23 elements (namely, As, Hg, Mn, Pb, Se, Tl, V, and Zn) exhibited significant differences between the two groups. In particular, the test showed significantly higher urinary levels of As (P50; 55.8 µg/g vs. 42.6 µg/g; *p* = 0.025), Hg (P50, 0.84 µg/g vs. 0.62 µg/g; *p* = 0.004), Mn (P50, 0.15 µg/g vs. 0.10 µg/g; *p* = 0.001), Pb (P50, 0.57 µg/g vs. 0.44 µg/g; *p* = 0.004), Se (P50, 26.3 µg/g vs. 23.3 µg/g; *p* = 0.023), Tl (P50, 0.46 µg/g vs. 0.30 µg/g; *p* < 0.001), V (P50, 0.34 µg/g vs. 0.24 µg/g; *p* = 0.033), and Zn (P50, 297 µg/g vs. 214 µg/g; *p* = 0.017) in exposed subjects when compared to controls.

**Figure 2:**
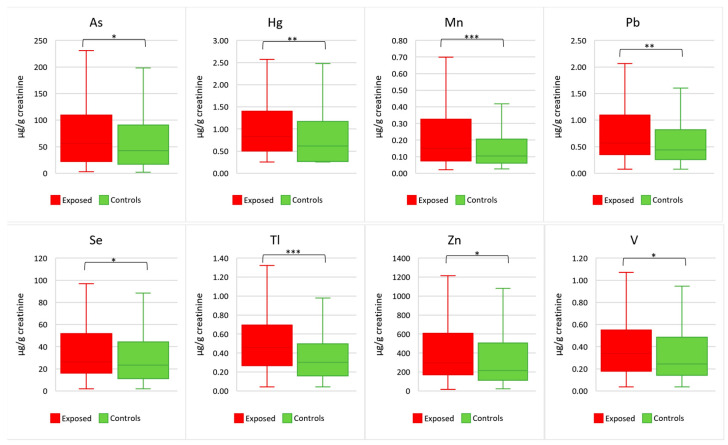
Box-plot of urinary elements (µg/g creatinine) significantly higher in exposed subjects compared to controls (* *p* < 0.05; ** *p*-value < 0.01; *** *p*-value < 0.001).

Figure 2 shows the box plots of the eight elements (As, Hg, Mn, Pb, Se, Tl, V, and Zn) that resulted significantly higher in the exposed subjects in comparison with controls.

**Figure 3:**
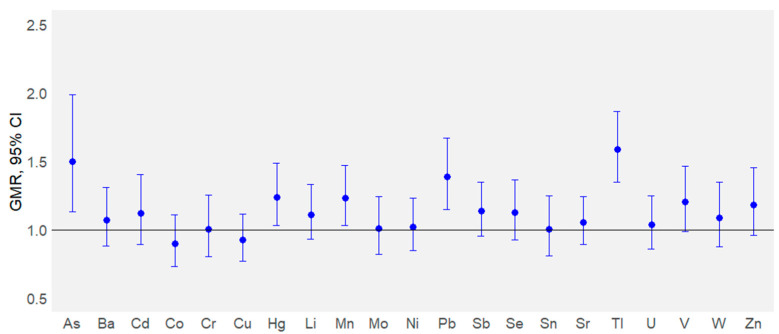
Relationship between urinary elements and Etna volcanic exposure expressed as geometric mean ratio (GMR) and 95% confidence intervals (95% CI).

Figure 3 reports the association between the element concentrations and the Etna volcanic area. Among the elements analyzed, the MLR analysis showed significantly increased concentrations of As (GMR, 1.50; 95% CI, 1.13–1.99), Hg (GMR, 1.24; 95% CI, 1.03–1.49), Mn (GMR, 1.23; 95% CI, 1.03–1.47), Pb (GMR, 1.39; 95% CI, 1.15–1.67), and Tl (GMR, 1.59; 95% CI, 1.35–1.87) in the exposed group. Specifically, an increase of 50% of As, 24% of Hg, 23% of Mn, 39% of Pb, and 59% of Tl in exposed subjects in comparison with the control ones was observed.

**Figure 4:**
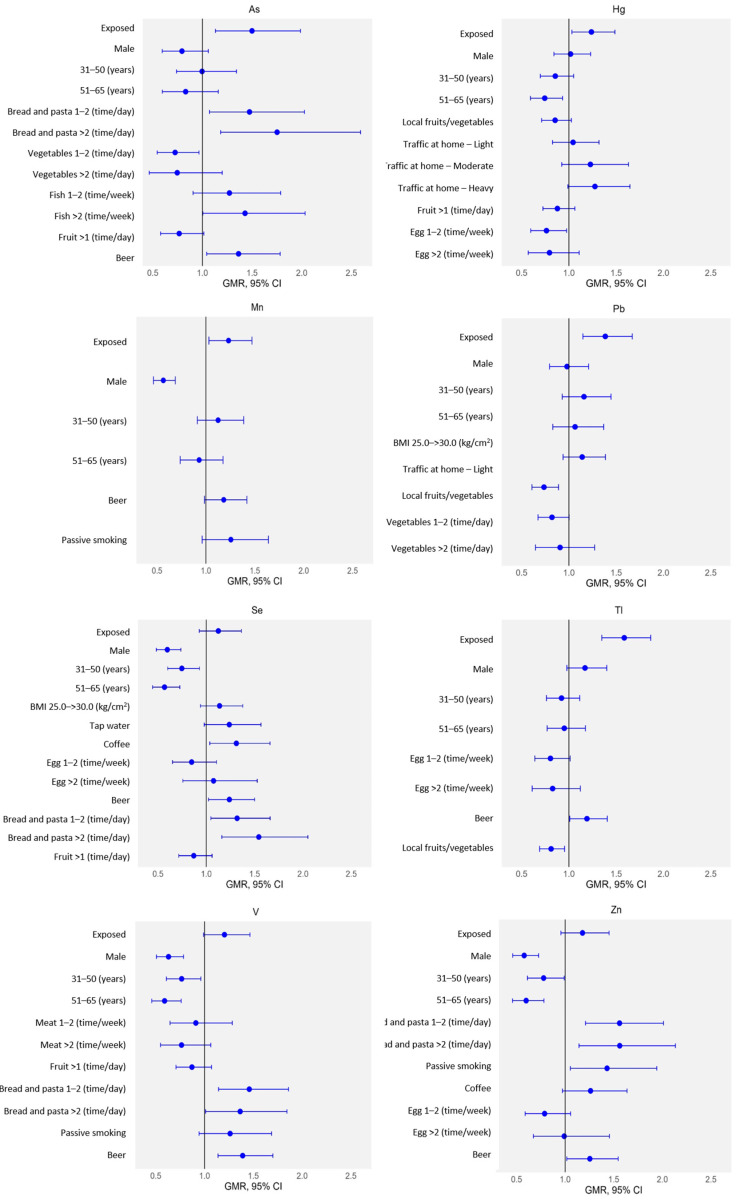
Association between urinary elements and influencing variables expressed as geometric mean ratio (GMR) and 95% confidence intervals (95% CI) by MLR analysis.

Figure 4 shows the associations between urinary levels of As, Hg, Mn, Pb, Se, Tl, V, and Zn and potential predictors by MLR analysis. It was confirmed that the volcanic activity primarily affected urinary levels of Hg, Mn, and Pb, whilst the significant increment of urinary As and Tl could be attributed to both the volcanic activity and other pathways of exposure. In particular, the intake of bread/pasta (1–2 times/day, GMR, 1.47; 95% CI, 1.07–2.03; >2 times/day, GMR, 1.75; 95% CI, 1.18–2.59), fish (>2 times/week, GMR, 1.43; 95% CI, 1.00–2.03), and beer (GMR, 1.36; 95% CI, 1.04–1.78) were significantly associated with higher As urinary concentration, and the intake of beer (GMR, 1.19; 95% CI, 1.01–1.41) significantly enhanced Tl urine level in subjects. Regarding Se, V, and Zn, the MLR analysis did not show the contribution of the Etna volcanic activity to these elements; whilst significant, other associations were observed. Specifically, the intake of coffee (GMR, 1.31; 95% CI, 1.04–1.66), beer (GMR, 1.24; 95% CI, 1.02–1.50), and bread/pasta (1–2 times/day, GMR, 1.32; 95% CI, 1.05–1.66; >2 times/day, GMR, 1.54; 95% CI, 1.16–2.05) significantly affected Se levels. Consumption of beer (GMR, 1.39; 95% CI, 1.14–1.70) and bread/pasta (1–2 times/day, GMR, 1.46; 95% CI, 1.14–1.86; >2 times/day, GMR, 1.37; 95% CI, 1.01–1.84) significantly influenced V levels. The intake of beer (GMR, 1.25; 95% CI, 1.02–1.57), bread/pasta (1–2 times/day, GMR, 1.56; 95% CI, 1.21–2.01; >2 times/day, GMR, 1.56; 95% CI, 1.14–2.13), and passive smoking (GMR, 1.43; 95% CI, 1.05–1.94) were associated with higher Zn levels.

The urinary concentrations of the remaining elements in exposed individuals appeared unaffected by Mt. Etna’s volcanic activity. Instead, their levels were predominantly influenced by extrinsic factors such as lifestyle patterns and dietary habits, including the consumption of specific foods and beverages. Table S3 (Supplementary Materials) reports the variables selected for inclusion in the MLR model. Levels of Cr were higher for the consumption of bread/pasta (>2 times/day, GMR, 1.43; 95% CI, 1.06–1.93), fish (>2 times/week, GMR, 1.37; 95% CI, 1.04–1.81), and beer (GMR, 1.25; 95% CI, 1.01–1.53). Copper content increased by consumption of tap water (GMR, 1.30; 95% CI, 1.04–1.62) and beer (GMR, 1.23; 95% CI, 1.04–1.60). Regarding Li, its urinary level was significantly associated with the consumption of bread/pasta (>2 times/day, GMR, 1.37; 95% CI, 1.05–1.78) and tap water (GMR, 1.30; 95% CI, 1.04–1.62). Beer consumption was associated with higher Mo concentrations (GMR, 1.25; 95% CI, 1.02–1.54). Levels of Ni increased significantly due to bread/pasta intake (>2 times/day, GMR, 1.36; 95% CI, 1.03–1.80), while Sb increased with tap water consumption (GMR, 1.24; 95% CI, 1.00–1.53). Higher levels were associated with tap water (GMR, 1.31; 95% CI, 1.02–1.69) and beer (GMR, 1.26; 95% CI, 1.02–1.55) for Sn. Uranium content increased with the intake of bread/pasta (>2 times/day, GMR, 1.32; 95% CI, 1.01–1.72). Concentration of W was associated with bread/pasta intake (1–2 times/day, GMR, 1.64; 95% CI, 1.26–2.13; >2 times/day, GMR, 1.51; 95% CI, 1.09–2.07) and beer (GMR, 1.25; 95% CI, 1.01–1.56).

The LDA model achieved an overall classification accuracy of 70.0% based on population group. Specifically, 81.3% of individuals in the control group and 56.9% of those in the exposed group were correctly classified. The LDA model identified Tl (*p* < 0.001) as the most discriminative element, followed by Mn (*p* = 0.037) and Hg (*p* = 0.042).

Table 2 presents the RVs for the study population, based on the median and P95 concentrations, and compares them with corresponding data from other countries, providing a broader context for interpreting environmental chemical exposure in the Sicilian population.

In addition, Table 3 compares the data in the studied population with the available HBGVs in a health risk assessment context, using the HBM-I and HBM-II values established by the German Human Biomonitoring Commission for urinary Cd, Hg, and Tl [29,30], and the Biomonitoring Equivalent (BE) values for urinary Ba, Bi, Cd, Mo, Se, and Sn [31,32,33,34,35,36,37]. The results showed that urinary concentrations of the analyzed elements were consistently below the established threshold values.

**Figure 5:**
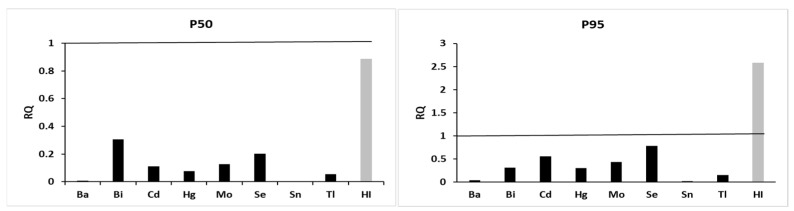
Risk of a mixture of seven trace elements in urine of the studied population at median (P50) and 95th percentile (P95); RQ: Risk Quotient; HI: Hazard Index.

Finally, the mixture risk assessment—in terms of HI and RQs—based on the available HBGVs was derived for both the median and P95 urinary metal concentrations of the Sicilian population and was calculated according to [28]. Figure 5 shows the RQs calculated on the median and P95 concentrations, and the HI as the sum of the individual RQs. None of the trace elements considered exceeded individual safe levels (RQ < 1), either at median or P95 values. The HI was equal to 0.89 at median values and 2.60 at P95 values, which is indicative that the mixture may pose a risk (HI > 1) only at the higher percentiles of exposure.

**Table 2:** European Reference Values (RVs) of elements in urine (µg/L) expressed as median (95th percentile).

Elements	Sicily (Italy) 2024 No. 360	Germany 2020 * No. 102	United Kingdom 2014 No. 132	Italy 2013 No. 394	Italy 2018 SIVR *** No. 200	Ireland 2011–2014 No. 100	Belgium 2010–2011 ** No. 1001	Switzerland 2009–2013 No. 903	France 2008–2010 No. 2000	Belgium 2007–2011 *** No. 204	France 2006–2007 No. 1991
	[this study]	[38]	[39]	[22]	[40]	[41]	[42]	[43]	[44]	[45]	[46]
As	35.1 (244)	10.3 (44)	10.48 (152.4)	18.2 (88.9)		9.8 (223.2) F 7.6 (69.4) M	14.1 (228)	11.8 (144)	16.0 (131)	17.2 (179.3)	12.68 (72.75)
Ba	1.31 (6.23)	2.9 (11)	1.37 (8.42)				1.91 (8.41)				
Be	<0.04 (<0.04)	<0.02 (<0.02)	0.0052 (0.0117)	0.16 (0.34)	<0.010 (0.034)		<0.007 (<0.007)	<0.002 (0.004)	0.01 (0.15)		
Bi	<0.11 (<0.11)	<0.01 (0.028)	<0.175 (<0.175)				<0.016 (0.042)	0.010 (0.095)			
Cd	0.11 (0.56)	0.23 (0.58)	0.13 (0.52)	0.71 (1.93)	0.255 (0.900)	0.5 (1.8) F 0.3 (0.9) M	0.276 (1.362)	0.186 (0.634)	0.42 (1.33)	0.239 (0.612)	0.32 (0.95)
Co	0.17 (1.11)	0.34 (1.3)	0.22 (1.04)	0.16 (0.56)	0.432 (2.24)		0.184 (1.281)	0.181 (0.972)	0.60 (1.89)		0.22 (1.40)
Cr	0.13 (0.87)	0.17 (0.35)	0.35 (0.79)	0.16 (0.44)	0.221 (0.600)	0.8 (1.5) F 0.8 (1.1) M	0.134 (0.524)	1.23 (2.48)	0.56 (1.60)		0.19 (0.65)
Cu	5.96 (22.5)	6.7 (15)	8.75 (9.30)	10.8 (26.6)		6.2 (27.1) F 7.3 (18.6) M	8.18 (23.9)	8.63 (21.7)			
Hg	0.53 (2.09)	0.25 (0.63)	0.43 (2.81)	1.35 (5.16)	11.6 (24.0)	0.4 (2.1) F 0.6 (2.5) M	0.382 (2.15)	0.249 (1.04)	1.08 (6.60)		
Li	18.7 (62.6)	27.6 (103)	11.0 (28.4)				22.9 (90.5)	21.6 (93.3)			
Mn	0.080 (0.47)	0.060 (0.12)	<0.092 (0.46)	0.12 (0.25)	0.289 (1.53)	0.2 (1.6) F 0.2 (0.9) M	<0.043 (0.560)	<0.568 (<0.568)	0.38 (1.07)		
Mo	26.3 (89.9)	30 (78)	29.13 (107.25)				31.3 (135)	26.6 (91.0)			
Ni	0.80 (3.68)	1.35 (4.0)	1.99 (6.35)	0.89 (3.04)	1.47 (4.44)		2.05 (5.64)	1.45 (6.27)	2.18 (5.99)		1.50 (4.54)
Pb	0.39 (1.74)	0.46 (1.0)	0.47 (7.63)		0.644 (2.64)	2.4 (4.2) F 2.2 (3.0) M	0.872 (3.45)	0.767 (2.29)	1.11 (3.76)		
Sb	0.028 (0.11)	0.030 (0.08)	<0.092 (0.26)	0.06 (0.18)	0.029 (0.095)		0.040 (0.314)	0.046 (0.265)	0.09 (0.41)		0.087 (0.320)
Se	18.3 (70.5)	14.1 (30)	13.4 (33.39)			15.5 (53.9) F 13.7 (39.7) M	25.1 (67.5)	17.3 (44.0)			
Sn	0.083 (0.45)	0.43 (1.2)	0.33 (2.44)	0.62 (1.99)			0.373 (3.565)	0.320 (1.50)			0.59 (2.81)
Sr	122 (338)	112 (286)	80.0 (350)								
Tl	0.27 (0.73)	0.34 (0.66)	0.17 (0.44)	0.27 (0.68)	0.203 (0.759)		0.211 (0.598)	0.158 (0.372)	0.25 (0.50)		
U	0.015 (0.048)	<0.008					<0.007 (0.043)				0.0049 (0.0212)
V	0.20 (1.15)	0.055 (0.13)	1.58 (3.79)	0.03 (0.11)	0.096 (0.855)		0.248 (1.265)	0.479 (0.966)	0.42 (1.21)		1.01 (2.79)
W	0.046 (0.31)	0.14 (0.38)	1.44 (6.04)								
Zn	185 (992)	247 (685)	180 (730)	389 (1146)			256 (1432)	216 (598)	348 (1039)		

* Mean; ** 97.5th Percentile; *** Geometric mean.

**Table 3:** Comparison with the Human Biomonitoring (HBM-I and HBM-II) values and Biomonitoring Equivalent (BE) values for urinary elements.

Elements	This Study (95th Percentile)	HBM-I	HBM-II	BE	Reference
Ba	6.23 µg/L 9.23 µg/g creatinine			192 µg/L (246 µg/g creatinine) ^a^	[36]
Bi	<0.11 µg/L			0.18 µg/L ^b^	[37]
Cd	0.56 µg/L	1 µg/L	4 µg/L	1.5 µg/L (2.0 µg/g creatinine) ^a^	[30,31]
	0.81 µg/g creatinine			1.2 µg/L (1.7 µg/g creatinine) ^c^	
Hg	2.09 µg/L	7 µg/L	25 µg/L		[30]
	3.52 µg/g creatinine	5 µg/g creatinine	20 µg/g creatinine		
Mo	89.9 µg/L			200–7500 µg/L ^a,c,d,e,f^	[34]
Se	70.5 µg/L			90–110 µg/L ^c,d^	[33]
Sn	0.45 µg/L 0.71 µg/g creatinine			20 µg/L (26 µg/g creatinine) ^e^	[35]
Tl	0.73 µg/L	5 µg/L			[30]

^a^ Calculated using the US EPA Reference Dose (RfD). ^b^ Calculated using the FDA Recommended Daily Intake (RDI). ^c^ Calculated using the ATSDR Chronic Minimal Risk Level (MRL). ^d^ Calculated using the IOM Upper Limit (UL). ^e^ Calculated using the RIVM Tolerable Daily Intake (TDI). ^f^ Calculated using the OECD SIDS 90-day toxicity study.

## 4. Discussion

### 4.1. Exposure to Etna Volcano

Active volcanoes can impact the environment and local ecosystems through multiple pathways: soil contamination from lava flows and ash deposits, atmospheric pollution from volcanic gases and fine particles, and water contamination when groundwater interacts with gases and minerals released by volcanic activity. These pathways enable the transfer of volcanic elements into locally grown plants and animals, potentially leading to biocontamination and health risks for nearby populations [17]. In this context, Mt. Etna is a significant source of volcanic emissions, including one of the world’s largest volcanic plumes. The fallout of ash and scoriae from Mt. Etna contains various metals and metalloids, which can contaminate the surrounding environment [16]. In addition, magma-derived CO_2_ emissions from Mt. Etna are substantial, contributing to lowering the pH of groundwater, creating acidic conditions that facilitate the leaching of metals such as Pb, Hg, and As from basaltic rocks [7]. After exsolution from magma, As, Hg, Mn, and V can be transported into groundwaters in different forms like Hg as vapour, and As, Mn, and V as gas species (manganese chloride, vanadium oxychloride, and arsenic sulfide) [47].

Given this context, the aim of this study was to assess whether chronic exposure to the recurrent emissions of gases and ashes from the Mt. Etna may result in elevated internal doses of trace elements among residents living in the surrounding area. To this end, urine samples were collected from exposed subjects living in the city of Catania and from individuals residing in distant areas from the volcano, serving as the control group (Figure 1). The Mann–Whitney results suggested that exposure to volcanic activity led to higher levels of urinary As, Hg, Mn, Pb, Se, Tl, V, and Zn in the exposed group compared to controls (Figure 2). The MLR analysis further supported the result that the increased urine levels of Hg, Mn, and Pb were primarily sourced from the volcanic activity (Figure 3), whilst the higher urine concentrations of As and Tl could be attributed to both volcanic activities and other sources, such as the consumption of specific foods (fish and bread/pasta consumption for As) and beverages (beer for both As and Tl) (Figure 4). Although the Mann–Whitney test suggested that Se, V, and Zn may originate from the volcanic source, the MRL results indicated that their urinary content was more strongly associated with specific foods or lifestyle-related factors. The LDA provided the identification of trace elements that most effectively differentiated exposed individuals from controls, thereby reinforcing the findings obtained through other statistical approaches. The lower accuracy observed for the exposed group may reflect a degree of overlap between the two populations, possibly due to shared environmental, dietary, or lifestyle factors, as also shown by the MLR analysis. Among the elements, Tl was the most influential discriminant element, followed by Hg and Mn. These findings support the hypothesis of volcanic activity contribution on the higher internal dose of selected trace elements in the exposed population compared to the control one. In the Etna environments, previous studies detected some trace elements at concentration levels higher than those found in other parts of Sicily. A study on Mt. Etna’s volcanic emissions found significant levels of As, Mn, Pb, Tl, V, and Zn in aerosols at the volcano’s rim and As and Tl levels in rainwater samples and the leaves of endemic plants [48]. Other studies using Sphagnum moss-bags to measure atmospheric deposition from Mt. Etna showed increased levels of As, Se, and Tl after volcanic emissions, as results of the volcanic gases’ condensation at various high- and low-temperature interactions in the volcanic plume [27]. Abbruzzo et al. [20] confirmed that volcanic environments in the Etnean region—including soils and groundwater—were enriched by As. When these soils are used for cereal cultivation and groundwater for irrigation, As can be taken up by plants through the phosphorus transport pathway, potentially making agricultural crops a significant source of As exposure for humans [20]. Barone et al. [6] assessed the human health risks of volcanic ash emissions from Mt. Etna, showing—through water leachate experiments—that As levels exceeded Italian limits for drinking water. A significant impact of Se emissions from Mt. Etna on human health was reported by Floor et al. [49]. By simulating rainwater–soil interaction using samples from Mt. Etna’s flanks and synthetic rain, the study identified this geogenic process as responsible for the natural mobilization of Se from volcanic sources into groundwater. Similarly, As and V were detected in Etna groundwaters at concentration levels generally higher than those commonly found in other Sicilian aquifers [7,47,50,51], while V concentration was found higher in tap water compared to mineral waters from other Italian sources [52,53]. Former studies in the Mt. Etna area showed the influence of volcanic emissions on trace element contents in food matrices and plants. Ferrante et al. [54] highlighted the presence of volcanic-origin metals in fruits and vegetables grown in the Mt. Etna area, suggesting a link between the enrichment of volcanogenic elements in soils and their uptake by plants through nutrient absorption pathways. Cimino et al. [55] found that elements of volcanic origin can accumulate in dairy products such as milk and cheese, as the mineral-rich volcanic soil influences the composition of grass and feed consumed by livestock, leading to the transfer of these elements into locally produced dairy goods. Varrica et al. [56] observed the accumulation of trace elements in lichens around Mt. Etna, with notably elevated levels of Pb and Zn compared to local substrates, identifying volcanic degassing as the primary source of this enrichment. Deposition of volcanic Hg in the local Etna environment was recognized by Martin et al. [57]. In this study, the accumulation of Hg in Castanea sativa (sweet chestnut) leaves growing in the Etna area was not influenced by soil concentrations, suggesting atmospheric re-emission or a groundwater leaching origin, and confirming Mt. Etna as a key environmental source of Hg [57]. Accordingly, Hg from Mt. Etna is primarily transported in its gaseous form (Hg^0^), accounting for approximately 7% of global non-eruptive Hg emissions from continuous volcanic degassing [58]. To our knowledge, the current literature reports only a few examples of the determination of trace elements in human matrices from people living close to an active volcanic area. The analysis of scalp hair to evaluate metal exposure in schoolchildren around Mt. Etna revealed enhanced intake of As, Mn, and V. Local rock composition and volcanic plume dispersion explain the differences, with water and local food being the main exposure pathways [19]. The higher levels of Mn detected in BAL samples from people exposed to Mt. Etna were attributed to volcanic emissions, based on comparisons with dust composition in southeastern Sicily [21].

### 4.2. Exposure to Other Variables

Beyond volcanic emissions, the study also highlighted the influence of environmental, dietary, and lifestyle factors on urinary trace element levels in the Sicilian population (Figure 4). Levels of As and Cr in urine increased significantly with fish consumption. Several forms of As may be present in urine, but, following fish consumption, the predominant species are the organic ones—such as arsenobetaine, arsenocholine, DMA, MMA, and arsenosugars—due to metabolic processing [59]. Thus, the As levels observed in the Sicilian population likely reflect the urinary excretion of As compounds resulting from seafood consumption occurring in the days prior to sample collection. Accordingly, fish and crustaceans from six Sicilian ports showed elevated As levels, with crustaceans exhibiting the highest concentrations [60]. Copat et al. [61] reported elevated Cr levels in fish from various Sicilian sites, with the highest level of concentration—up to 1 µg/g—near industrial areas, nearly 10 times higher than in fish from unpolluted zones. Bonsignore et al. [62] found a heterogeneous distribution of As, Cd, Cr, Cu, Hg, Ni, Pb, and Zn in the edible tissues of marine species from the Tuscany coast, attributed mainly to industrial activity. Similarly, Sepe et al. [63] detected Cd, Cr, Pb, and V levels in six fish species from the Adriatic coast, although with low Cr levels. Urinary levels of As, Cr, Li, Ni, Se, U, V, W, and Zn increased significantly due to bread and pasta consumption. The presence of As in soils and groundwater used for cultivating cereals in Etna may transfer As to plant tissues through the phosphorus channel, making crops a source of As exposure [20]. Many trace elements as Zn, Cr, Cu, Pb, and Cd were detected in the soil, roots, and shoots of Italian wheat samples, with Cu, Zn, Cd, Cr, Pb, and As translocating into the wheat plant [64,65]. Olivieri et al. [66] analyzed heavy metals in durum wheat and pasta produced using an ancient Sicilian millstone. They found that concentrations of As, Cd, Hg, and Pb were below European limits, indicating the products are safe for consumption [66]. The concentration of elements such as As, Cr, Cu, Mo, Se, Sn, Tl, V, W, and Zn in urine was also significantly influenced by beer consumption, suggesting regular intake as a notable source of exposure. The presence of metals in beer may result from various sources and stages throughout the brewing process, including raw materials, additives, processing aids, packaging, and equipment. These components can leach metals into the product due to the moderately acidic pH of beer (~4.2). Food-grade diatomaceous earth, used as a filtration aid, can significantly increase As levels in the final product [67], while V content in diatomite correlates directly with its concentration in filtered beer [68]. Elevated urinary Zn levels observed in beer consumers are consistent with Zn’s role in yeast metabolism during fermentation [69]. Furthermore, a study on Se biofortification in cereals demonstrated that the use of sodium selenate fertilizer led to an almost six-fold increase in Se concentration in beer, compared to beer produced from barley grown without fertilization [70]. Urinary levels of Cu, Li, Sb, and Sn increased significantly with tap water consumption. Dinelli et al. [53] analyzed 157 tap water samples from various Italian regions, including Sicily, and reported that the presence of trace elements reflected multiple factors, such as geological characteristics, geographic location, and the water distribution system. Specifically, Cu and Sn were associated with the distribution infrastructure, Li with coastal areas and volcanic formation, and Sb with mineralized veins [53]. Russo et al. [71] examined 13 tap water samples from Campania (South Italy), and found very low levels of Hg, Pb, Cr, Co, Ni, Cd, and As. Additionally, Sorlini et al. [72] identified water stagnation and the quality of piping materials as significant contributors to the contamination of Ni, Pb, and Zn in tap water samples from public buildings in Lombardy, Northern Italy. Another result was the higher urine levels of Zn associated with passive smoking. Second-hand smoke has been identified as a potential source of increased Zn exposure. Scientific evidence associated tobacco smoke exposure with elevated salivary Zn levels in children [73], while passive smoke exposure correlated with Zn concentration in the dental tissues of children, suggesting a chronic accumulation of the metal [74]. In this context, cigarettes have been reported to contain relatively high concentrations of Zn, with approximately 70% of the metal being transferred to the smoke phase. This Zn may be subsequently inhaled by both the active smoker and those nearby [75]. Another variable that positively influenced the urinary content of Se was the consumption of coffee. Regarding the accumulation of metals in coffee plants and beans, environmental factors such as soil, climate, and water are expected to play a key role in the elemental composition throughout the plants’ and beans’ life cycle. Previous studies reported relatively low levels of Se in coffee beans originating from various regions, including Brazil, Kenya, India, and the Caribbean [76,77,78].

### 4.3. Reference Values (RVs), Health-Based Guidance Values (HBGVs), and Risk Quotients (RQs)

Reference values (RVs) for environmental chemicals, based on the IFCC and IUPAC reference interval concepts, indicate background exposures in the general population [79,80]. Establishing RVs requires a reference population that meets specific criteria, such as a large sample size, defined exclusion and partitioning criteria, and high-quality analytical methods [40,81,82,83,84]. In this study, all these criteria were fulfilled, allowing the obtained HBM data to be considered as representative RVs for urinary element concentrations in the Sicilian population. This study also compared the RVs with those reported in other HBM studies carried out in various countries to assess whether the observed exposure was higher than usual. Examples of RVs are those from the Irish EuroMOTOR project [41], the French ENNS survey [46], the French IMEPOGE study [44], the Belgian FLESH-II study [45], the Swiss SKiPOGH study [43], the Italian Society of Reference Values [40], the Italian SPoTT study [22], and the HBM studies from Belgium [42], Germany [38], and the United Kingdom [39]. European HBM activities have often been fragmented, making cross-study comparisons challenging due to differences in protocols and methodologies. Despite these limitations, the RVs observed in the Sicilian population were generally comparable to, or even lower than, those reported in other HBM studies for all analyzed elements (Table 2). These findings suggest that, despite regional variations in environmental exposure, dietary habits, and lifestyles, the exposure levels to trace elements in the Sicilian population are in line with European averages. The comparison between the data obtained in this study and those reported in previous research conducted in Italy revealed a substantial consistency in the results, or even a decrease in trace element levels in the urine of the Italian population [22,40]. These findings suggest a possible improvement in environmental conditions, as well as the effectiveness of regulatory interventions and more sustainable management practices implemented over the years in Italy. Moreover, the use of HBGVs, including HBM-I and HBM-II, can facilitate the interpretation of population-level HBM data within the context of health risk assessment. These thresholds, established by the German Human Biomonitoring Commission, are available for selected substances including urinary Cd, Hg, and Tl, and serve as benchmarks for evaluating potential health risks associated with internal exposure levels. HBM-I indicates a biomarker concentration below which no adverse health effects are expected, while HBM-II is an action level above which adverse health effects may occur, necessitating immediate action to reduce exposure [29,30]. Another useful indicator is the BE value which aligns with HBGVs [31,32,33,34,35,36,37]. Element concentrations below their respective BE values indicate a low priority for public health concern, whereas values exceeding BEs suggest medium-to-high priority and warrant further investigation into exposure pathways and influencing factors. In this context, urinary levels of Ba, Bi, Cd, Hg, Mo, Se, Sn, and Tl in the Sicilian population were consistently below the HBM-I, HBM-II, and BE thresholds (Table 3). Consequently, no urgent measures were deemed necessary to mitigate exposure to these eight elements. Additionally, the mixture risk assessment—in terms of HI and RQs—was derived from both the median and P95 concentrations using the available HBGVs (Figure 5). Details on the mixture risk assessment methodology are reported in [28]. Due to the limited availability of HBGVs, the mixture risk assessment was conducted for eight trace elements (Ba, Bi, Cd, Hg, Mo, Se, Sn, and Tl). The results indicated that exposure to each individual element did not pose a health risk, as all RQ values were below 1 at both the median and P95 concentrations. Similarly, no risk was observed for the combined exposure at the median scenario (HI < 1 at median). However, under the worst-case scenario (HI > 1 at P95), potential health risks from the mixture cannot be excluded. These findings should be interpreted with caution, given the limited number of trace elements included in the assessment due to the lack of available HBGVs and the associated uncertainties.

## 5. Conclusions

Mt. Etna, an active volcano characterized by frequent emissions of gases, ash, and occasional lava flows, presents a unique environmental exposure scenario for nearby populations. Adults and children residing in its vicinity are chronically exposed to volcanic emissions, even during quiescent phases, potentially leading to the internal dose of trace elements through various exposure pathways, including inhalation and ingestion. To evaluate the extent of this exposure, an HBM campaign was conducted to quantify 23 trace elements in the urine of individuals living near Mt. Etna, compared to control subjects from other areas of Sicily. The results revealed significantly higher urinary concentrations of As, Hg, Mn, Pb, and Tl in the exposed group. Among these, Hg, Mn, and Pb levels appeared to be primarily influenced by volcanic activity, while the increases in As and Tl were likely due to a combination of volcanic emissions and additional exposure predictors. While several associations between internal doses of elements and exposure predictors are confirmed by the existing literature, others were newly identified and involved trace elements that have received limited attention to date. Moreover, the complete dataset was used to derive the RVs for the measured elements as indicators of background exposure levels in the Sicilian population. The RVs for the studied population—despite their residence in a volcanically active region—were aligned with those reported in other Italian and European studies and remained within health-based safety thresholds, indicating no elevated risk from trace element exposure. In conclusion, HBM proved to be an effective tool for assessing trace element exposure, offering valuable reference data for national and international comparisons and supporting the identification of at-risk populations to inform evidence-based public health policies and preventive strategies.

## Figures and Tables

**Table 1:** Urine element concentrations (µg/g creatinine) expressed as median (P50) and 5th–95th percentile (P5–P95); bolded values highlight statistically significant increases in the exposed group.

Elements	Total Population	Exposed	Controls	*p*-Value ^1^
	P50 (P5–P95)	P50 (P5–P95)	P50 (P5–P95)	
**As**	47.9 (5.21–337)	**55.8 (7.70–434)**	**42.6 (4.30–254)**	**0.025**
Ba	1.74 (0.38–9.23)	1.81 (0.47–9.96)	1.59 (0.29–9.03)	0.444
Be	<LoD	<LoD	<LoD	-
Bi	<LoD	<LoD	<LoD	-
Cd	0.15 (0.031–0.81)	0.17 (0.017–0.87)	0.140 (0.026–0.776)	0.058
Co	0.23 (0.053–1.66)	0.23 (0.044–1.49)	0.23 (0.055–1.69)	0.560
Cr	0.18 (0.054–1.42)	0.20 (0.053–1.42)	0.17 (0.056–1.33)	0.506
Cu	7.83 (2.05–36.9)	8.37 (2.18–34.0)	7.47 (2.04–37.7)	0.319
**Hg**	0.73 (0.25–3.52)	**0.84 (0.25–4.14)**	**0.62 (0.25–3.16)**	**0.004**
Li	26.8 (6.29–91.3)	28.6 (6.87–83.8)	25.3 (5.34–92.5)	0.362
**Mn**	0.11 (0.040–0.62)	**0.15 (0.38–0.68)**	**0.10 (0.037–0.50)**	**0.001**
Mo	37.3 (5.43–140)	38.9 (7.27–139)	35.3 (4.94–141)	0.355
Ni	1.06 (0.25–5.02)	1.14 (0.25–4.33)	0.99 (0.26–5.78)	0.338
**Pb**	0.52 (0.13–2.96)	**0.57 (0.18–3.38)**	**0.44 (0.079–2.42)**	**0.004**
Sb	0.038 (0.010–0.18)	0.042 (0.014–0.21)	0.034 (0.010–0.16)	0.055
**Se**	24.8 (5.78–115)	**26.3 (5.99–112)**	**23.3 (5.50–120)**	**0.023**
Sn	0.11 (0.025–0.71)	0.12 (0.025–0.85)	0.10 (0.025–0.60)	0.190
Sr	163 (46.8–599)	165 (54.9–552)	160 (36.4–624)	0.531
**Tl**	0.36 (0.085–1.13)	**0.46 (0.12–1.27)**	**0.30 (0.044–0.99)**	**<0.001**
U	0.020 (0.008–0.078)	0.020 (0.008–0.087)	0.020 (0.008–0.066)	0.737
**V**	0.29 (0.070–1.71)	**0.34 (0.071–1.94)**	**0.24 (0.070–1.30)**	**0.033**
W	0.063 (0.019–0.45)	0.070 (0.19–0.48)	0.057 (0.019–0.43)	0.098
**Zn**	258 (53.1–1509)	**297 (65.2–1388)**	**214 (41.1–1550)**	**0.017**

^1^ *p*-values obtained by the Mann–Whitney test of exposed vs. control subjects. Be: LoD < 0.04 µg/mL; Bi: LoD < 0.11 µg/mL.

## Data Availability

The datasets presented in this article are not available because of privacy or ethical reasons.

## Supplementary Materials

The following supporting information can be downloaded at https://www.mdpi.com/article/10.3390/toxics13121080/s1, Table S1: Characteristics of the subjects; Table S2: Performances of the inductively coupled plasma mass spectrometry (ICP-MS) method; Table S3: Predictors included in the MRL model, with bold text indicating significant positive predictors.
