# Supplementary material for: Human Exposure to Metals and Potential Human Health Risk in a Volcanic Environment in Italy
Source: Toxics. 2025 Dec 15;13(12):1080. doi: 10.3390/toxics13121080 (PMC12737141; doi:10.3390/toxics13121080)
Supplement: Supplementary file 1 [file toxics-13-01080-s001.zip › toxics-4014217-supplementary.pdf]

Supplementary materials

Human Exposure to Metals and Potential Human Health Risk in a Volcanic Environment in Italy

Giovanni Forte [1,†], Venerando Rapisarda [2,†], Flavia Ruggieri [1], Beatrice Battistini [1], Lisa Bauleo [1], Veronica Filetti [2], Elena Grignani [3], Piero Lovreglio [4], Serena Matera [2], Paola Senia [2], Francesca Vella [5], Ermanno Vitale [5],
Beatrice Bocca [1,*,‡] and Ivo Iavicoli [6,7,‡]

[1] Department of Environment and Health, Italian National Institute of Health, 00161 Rome, Italy; giovanni.forte@iss.it (G.F.); flavia.ruggieri@iss.it (F.R.); beatrice.battistini@iss.it (B.Ba.); lisa.bauleo@iss.it (L.B.); beatrice.bocca@iss.it (B.Bo)
[2] Department of Clinical and Experimental Medicine, University of Catania, 95124 Catania, Italy; venerando.rapisarda@unict.it (V.R.); verofiletti@gmail.com (V.F.); serena.matera@yahoo.it (S.M.); paosenia@hotmail.it (P.S.)
[3] Environmental Research Center, Istituti Clinici Scientifici Maugeri IRCCS, 27100 Pavia, Italy; elena.grignani@icsmaugeri.it
[4] Interdisciplinary Department of Medicine, University of Bari, 70124 Bari, Italy; piero.lovreglio@uniba.it
[5] Department of Medicine and Surgery, University of Enna "Kore", 94100 Enna, Italy; francescav.89@libero.it (F.V.); ermannovitale@gmail.com (E.V.)
[6] Department of Healthcare Surveillance and Bioethics, Catholic University of Sacred Heart, 00168 Rome, Italy; ivo.iavicoli@unicatt.it (I.I.)
[7] Fondazione Policlinico Universitario A. Gemelli IRCCS, 00168 Rome, Italy.
* Correspondence: beatrice.bocca@iss.it
† These authors contributed equally to this work.
‡ These authors contributed equally to this work.

Table S1. Characteristics of the subjects

Variable	Category	Total population (no. 360)	Exposed (no. 167)	Controls (no. 193)
Gender	Female	115	66	49
	Male	245	101	144
Age (years)	20-30	104	48	56
	31-50	158	72	86
	51-65	98	47	51
BMI (kg/cm^2)	<18.0-24.9	199	95	104
	25.0- >30.0	161	72	89
Type of water	Tap	72	49	23
	Bottled	288	118	170
Coffee	No	69	21	48
	Yes	291	146	145
Wine	No	199	88	111
	Yes	161	79	82
Beer	No	207	97	110
	Yes	153	70	83
Fish (time/week)	< 1	90	59	31
	1-2	170	42	128
	>2	100	66	34
Meat (time/week)	< 1	38	21	17
	1-2	127	54	73
	>2	195	92	103
Egg (time/week)	< 1	51	26	25
	1-2	261	111	150
	>2	48	30	18
Bread and pasta (time/day)	< 1	82	51	31
	1-2	198	91	107
	>2	80	25	55
Vegetables (time/day)	< 1	161	74	87
	1-2	166	73	93
	>2	33	20	13
Fruit (time/day)	< 1	129	58	71
	>1	231	109	122
Local fruits/vegetables	No	200	84	116
	Yes	160	83	77
Mushroom	No	215	95	120
	Yes	145	72	73
Passive smoking	No	315	141	174
	Yes	45	26	19
Traffic at home	No	168	47	121
	Light	87	57	30
	Moderate	60	31	29
	Heavy	45	32	13
Traffic at work	No	182	58	124
	Light	37	24	13
	Moderate	94	54	40
	Heavy	47	31	16

Table S2. Performances of the inductively coupled plasma mass spectrometry (ICP-MS) method

Elements	Analytical mass	Analytical mode	Interferences	LoD µg/L	Samples < LoD in % (no. 360)	Seronorm Trace Elements Urine L-1		Seronorm Trace Elements Urine L-2	
						Accuracy µg/L (%)	Precision (%)	Accuracy µg/L (%)	Precision (%)
**As**	75	KED	$^{40}Ar^{35}Cl$	0.92		97 (102)	4.5	198 (101)	0.8
**Ba**	138	STD		0.06		3.60* (94)	7.2	21.5* (98)	1.5
**Be**	9	STD		0.04	100	<0.005 (LoD)	nd	5.4 (95)	2.2
**Bi**	209	STD		0.11	100	0.010 (<LoD)	nd	19.8 (97)	1.7
**Cd**	114	STD		0.02		0.062 (94)	7.9	4.7 (100)	3.5
**Co**	59	KED	$^{43}Ca^{16}O$; $^{40}Ar^{19}F$	0.03		0.19 (95)	5.5	12.8 (97)	2.4
**Cr**	52	KED	$^{40}Ar^{12}C$	0.05	4.17	7.7 (101)	2.8	30.4 (100)	2.5
**Cu**	63	KED	$^{40}Ar^{23}Na$	1.40		26 (98)	4.1	68 (94)	1.0
**Hg**	202	STD		0.40	19.7	1.11 (107)	3.6	41.5 (99)	2.0
**Li**	7	STD		1.87		5.20* (102)	4.0	5.20* (97)	3.4
**Mn**	55	KED	$^{39}K^{16}O$	0.03		0.33 (103)	6.6	9.3 (101)	3.6
**Mo**	100	STD		1.44		17.0 (105)	1.8	73.8 (95)	1.1
**Ni**	60	KED	$^{44}Ca^{16}O$; $^{36}Ar^{24}Mg$	0.12		1.00 (98)	5.4	38.4 (96)	2.8
**Pb**	208	STD		0.13	3.33	1.51 (107)	7.6	78.4 (94)	0.9
**Sb**	123	STD		0.015	5.00	2.3 (102)	5.1	105 (100)	0.7
**Se**	82	KED	$^{40}Ar^{42}Ca$; $^{66}Zn^{16}O$	2.00		10.5 (100)	2.9	66.2 (97)	1.5
**Sn**	120	STD		0.04	7.50	2.4 (96)	3.7	46.0 (99)	1.2
**Sr**	88	KED	$^{36}Ar^{52}Cr$;$^{40}Ar^{48}Ti$; $^{40}Ar^{48}Ca$	7.16		74.0* (99)	1.0	76.0* (98)	1.8
**Tl**	205	STD		0.07	3.89	0.106 (109)	7.0	8.4 (100)	2.7
**U**	238	STD		0.01	39.2	0.017 (93)	7.2	0.022 (94)	3.9
**V**	51	KED	$^{35}Cl^{16}O$	0.05		0.13 (95)	6.3	23.1 (99)	3.3
**W**	184	STD		0.03	27.2	nr	nd	nr	nd
**Zn**	64	KED	$^{40}Ar^{26}Mg$	17.1		171 (101)	1.1	1179 (101)	0.5

STD: standard; KED: kinetic energy discrimination; LoD: limit of detection; nr: not reported; nd: not detected

*Elements reported as informative values only.

Table S3. Predictors included in the MRL model, with bold text indicating significant positive predictors

Elements	Variable	Category	*β coefficient*	95% C.I.			*p* value
As	Group	Controls	Ref.				
		Exposed	0.405	0.124	–	0.687	**0.005**
	Bread and pasta (time/day)	<1	Ref.				
		1-2	0.388	0.069	–	0.707	**0.017**
		> 2	0.561	0.169	–	0.953	**0.005**
	Vegetables (time/day)	<1	Ref.				
		1-2	-0.321	-0.607	–	-0.036	0.028
		> 2	-0.292	-0.766	–	0.182	0.226
	Fish (time/week)	<1	Ref.				
		1-2	0.241	-0.099	–	0.581	0.164
		> 2	0.357	0.005	–	0.710	**0.047**
	Fruit (time/day)	<1	Ref.				
		>1	-0.266	-0.546	–	0.014	0.063
	Beer	No	Ref.				
		Yes	0.310	0.043	–	0.578	**0.023**
Ba	Group	Controls	Ref.				
		Exposed	0.070	-0.128	–	0.268	0.489
	BMI (kg/cm^2)	<18.0-24.9	Ref.				
		25.0->30.0	0.189	-0.017	–	0.394	0.072
	Local fruits/vegetables	No	Ref.				
		Yes	-0.338	-0.536	–	-0.141	0.001
Cd	Group	Controls	Ref.				
		Exposed	0.112	-0.116	–	0.340	0.334
	BMI (kg/cm^2)	<18.0-24.9	Ref.				
		25.0->30.0	0.163	-0.048	–	0.374	0.129
	Fish (time/week)	<1	Ref.				
		1-2	0.032	-0.238	–	0.302	0.817
		> 2	-0.211	-0.482	–	0.060	0.127
	Bread and pasta (time/day)	<1	Ref.				

		1-2	0.171	-0.086	–	0.428	0.191
		> 2	0.308	-0.009	–	0.624	0.057
	Beer	No	Ref.				
		Yes	0.195	-0.013	–	0.404	0.066
	Egg (time/week)	<1	Ref.				
		1-2	-0.321	-0.613	–	-0.028	0.032
		> 2	-0.188	-0.580	–	0.204	0.345
	Meat (time/week)	<1	Ref.				
		1-2	-0.098	-0.467	–	0.270	0.600
		> 2	-0.279	-0.638	–	0.080	0.127
	Fruit (time/day)	<1	Ref.				
		>1	-0.152	-0.369	–	0.064	0.168
Co	Group	Controls	Ref.				
		Exposed	-0.105	-0.313	–	0.103	0.320
	Bread and pasta (time/day)	<1	Ref.				
		1-2	0.230	-0.050	-	0.519	0.128
		>2	0.235	-0.075	-	0.544	0.137
Cr	Group	Controls	Ref.				
		Exposed	0.002	-0.222	–	0.227	0.984
	Bread and pasta (time/day)	<1	Ref.				
		1-2	-0.018	-0.266	–	0.230	0.885
		> 2	0.359	0.056	–	0.662	**0.020**
	Type of water	Bottled	Ref.				
		Tap	0.196	-0.065	–	0.456	0.140
	Fish (time/week)	<1	Ref.				
		1-2	0.145	-0.118	–	0.408	0.279
		> 2	0.314	0.036	–	0.593	**0.027**
	Local fruits/vegetables	No	Ref.				
		Yes	-0.244	-0.450	-	-0.037	0.021
	Beer	No	Ref.				
		Yes	0.220	0.013	–	0.427	**0.037**
	Group	Controls	Ref.				

		Exposed	-0.076	-0.259	–	0.106	0.411
	Egg (time/week)	<1	Ref.				
		1-2	-0.317	-0.567	–	-0.068	0.013
		> 2	-0.202	-0.531	–	0.126	0.227
	Type of water	Bottled	Ref.				
Cu		Tap	0.264	0.043	–	0.484	**0.019**
	Coffee	No	Ref.				
		Yes	0.207	-0.016	–	0.429	0.069
	Passive smoking	No	Ref.				
		Yes	0.175	-0.086	–	0.436	0.188
	Beer	No	Ref.				
		Yes	0.210	0.032	–	0.389	**0.021**
Hg	Group	Controls	Ref.				
		Exposed	0.214	0.031	–	0.397	**0.022**
	Local fruits/vegetables	No	Ref.				
		Yes	-0.158	-0.341	-	0.026	0.092
	Traffic at home	No	Ref.				
		Light	0.043	-0.190	–	0.277	0.717
		Moderate	0.205	-0.079	–	0.489	0.156
		Heavy	0.243	-0.011	–	0.498	0.061
	Fruit (time/day)	<1	Ref.				
		>1	-0.130	-0.321	–	0.061	0.183
	Egg (time/week)	<1	Ref.				
		1-2	-0.270	-0.517	–	-0.024	0.032
		> 2	-0.230	-0.563	–	0.103	0.176
Li	Group	Controls	Ref.				
		Exposed	0.105	-0.074	–	0.284	0.248
	Bread and pasta (time/day)	<1	Ref.				
		1-2	0.167	-0.047	–	0.382	0.126
		> 2	0.313	0.047	–	0.579	**0.021**
	Type of water	Bottled	Ref.				
		Tap	0.259	0.038	–	0.479	**0.022**

	Local fruits/vegetables	No	Ref.				
		Yes	-0.312	-0.490	–	-0.133	0.001
	Vegetables (time/day)	<1	Ref.				
		1-2	-0.223	-0.411	–	-0.036	0.020
		> 2	-0.171	-0.485	–	0.143	0.284
	Beer	No	Ref.				
		Yes	0.148	-0.034	–	0.331	0.110
Mn	Group	Controls	Ref.				
		Exposed	0.208	0.030	–	0.386	**0.022**
	Beer	No	Ref.				
		Yes	0.167	-0.015	–	0.350	0.072
	Passive smoking	No	Ref.				
		Yes	0.227	-0.040	–	0.494	0.095
Mo	Group	Controls	Ref.				
		Exposed	0.009	-0.199	–	0.217	0.930
	Mushrooms	No	Ref.				
		Yes	0.144	-0.064	–	0.351	0.173
	Type of water	Bottled	Ref.				
		Tap	0.210	-0.047	–	0.467	0.110
	Beer	No	Ref.				
		Yes	0.227	0.019	–	0.434	**0.032**
	Egg (time/week)	<1	Ref.				
		1-2	-0.273	-0.561	–	0.015	0.063
		> 2	-0.133	-0.518	–	0.251	0.495
Ni	Group	Controls	Ref.				
		Exposed	0.021	-0.164	–	0.207	0.821
	Beer	No	Ref.				
		Yes	0.152	-0.036	–	0.339	0.113
	Bread and pasta (time/day)	<1	Ref.				
		1-2	0.220	-0.007	–	0.447	0.058
		> 2	0.308	0.031	–	0.586	**0.030**
Pb	Group	Controls	Ref.				

		Exposed	0.326	0.138	–	0.513	**0.001**
	BMI (kg/cm^2)	<18.0-24.9	Ref.				
		25.0->30.0	0.131	-0.064	–	0.327	0.186
	Local fruits/vegetables	No	Ref.				
		Yes	-0.306	-0.495	–	-0.116	0.002
	Vegetables (time/day)	<1	Ref.				
		1-2	-0.197	-0.396	–	0.002	0.053
		> 2	-0.098	-0.437	–	0.241	0.571
Sb	Group	Controls	Ref.				
		Exposed	0.126	-0.046	–	0.298	0.150
	Bread and pasta (time/day)	<1	Ref.				
		1-2	-0.122	-0.328	–	0.084	0.244
		> 2	0.110	-0.145	–	0.364	0.397
	Type of water	Bottled	Ref.				
		Tap	0.214	0.004	–	0.425	**0.046**
	Local fruits/vegetables	No	Ref.				
		Yes	-0.143	-0.312	–	0.027	0.100
	Vegetables (time/day)	<1	Ref.				
		1-2	-0.238	-0.417	–	-0.060	0.009
		> 2	0.150	-0.149	–	0.449	0.325
Se	Group	Controls	Ref.				
		Exposed	0.118	-0.075	–	0.310	0.230
	BMI (kg/cm^2)	<18.0-24.9	Ref.				
		25.0->30.0	0.129	-0.062	–	0.320	0.185
	Type of water	Bottled	Ref.				
		Tap	0.214	-0.021	–	0.449	0.075
	Coffee	No	Ref.				
		Yes	0.271	0.036	–	0.507	**0.024**
	Egg (time/week)	<1	Ref.				
		1-2	-0.164	-0.429	–	0.100	0.223
		> 2	0.073	-0.278	–	0.423	0.684
	Beer	No	Ref.				

		Yes	0.214	0.023	–	0.406	**0.028**
	Bread and pasta (time/day)	<1	Ref.				
		1-2	0.277	0.046	–	0.507	**0.019**
		> 2	0.434	0.149	–	0.719	**0.003**
	Fruit (time/day)	<1	Ref.				
		>1	-0.139	-0.336	–	0.058	0.167
Sn	Group	Controls	Ref.				
		Exposed	0.005	-0.209	–	0.220	0.962
	Type of water	Bottled	Ref.				
		Tap	0.271	0.018	–	0.523	**0.036**
	Egg (time/week)	<1	Ref.				
		1-2	-0.264	-0.548	–	0.021	0.069
		> 2	-0.204	-0.587	–	0.180	0.297
	Fruit (time/day)	<1	Ref.				
		>1	-0.283	-0.493	–	-0.072	0.009
	Beer	No	Ref.				
		Yes	0.229	0.021	–	0.436	**0.031**
Sr	Group	Controls	Ref.				
		Exposed	0.053	-0.111	–	0.217	0.527
	Bread and pasta (time/day)	<1	Ref.				
		1-2	0.181	-0.020	–	0.382	0.078
		> 2	0.171	-0.077	–	0.419	0.175
	Egg (time/week)	<1	Ref.				
		1-2	-0.223	-0.453	–	0.008	0.058
		> 2	-0.282	-0.591	–	0.027	0.074
	Local fruits/vegetables	No	Ref.				
		Yes	-0.208	-0.372	–	-0.043	0.014
	Vegetables (time/day)	<1	Ref.				
		1-2	-0.115	-0.289	–	0.059	0.196
		> 2	0.146	-0.153	–	0.445	0.337
	Beer	No	Ref.				
		Yes	0.119	-0.050	–	0.287	0.168

Tl	Group	Controls	Ref.				
		Exposed	0.461	0.298	–	0.623	**0.000**
	Egg (time/week)	<1	Ref.				
		1-2	-0.217	-0.447	–	0.013	0.065
		> 2	-0.189	-0.492	–	0.115	0.222
	Beer	No	Ref.				
		Yes	0.175	0.009	–	0.341	**0.039**
	Local fruits/vegetables	No	Ref.				
		Yes	-0.209	-0.370	–	-0.047	0.012
U	Group	Controls	Ref.				
		Exposed	0.035	-0.153	–	0.223	0.714
	Bread and pasta (time/day)	<1	Ref.				
		1-2	0.104	-0.120	–	0.328	0.361
		> 2	0.275	0.007	–	0.543	**0.044**
	Local fruits/vegetables	No	Ref.				
		Yes	-0.133	-0.312	–	0.045	0.143
V	Group	Controls	Ref.				
		Exposed	0.186	-0.011	–	0.383	0.064
	Meat (times/week)	<1	Ref.				
		1-2	-0.093	-0.437	–	0.250	0.593
		> 2	-0.269	-0.600	–	0.062	0.110
	Fruit (times/day)	<1	Ref.				
		>1	-0.138	-0.346	–	0.070	0.191
	Bread and pasta (time/day)	<1	Ref.				
		1-2	0.377	0.134	–	0.621	**0.002**
		> 2	0.311	0.010	–	0.612	**0.043**
	Passive smoking	No	Ref.				
		Yes	0.233	-0.058	–	0.523	0.116
	Beer	No	Ref.				
		Yes	0.330	0.129	–	0.531	**0.001**
W	Group	Controls	Ref.				
		Exposed	0.081	-0.135	–	0.297	0.461

	Type of water	Bottled	Ref.				
		Tap	0.202	-0.064	–	0.468	0.137
	Bread and pasta (time/day)	<1	Ref.				
		1-2	0.494	0.234	–	0.755	**0.000**
		> 2	0.410	0.089	–	0.730	**0.012**
	Beer	No	Ref.				
		Yes	0.227	0.012	–	0.442	**0.039**
Zn	Group	Controls	Ref.				
		Exposed	0.164	-0.045	–	0.373	0.123
	Bread and pasta (time/day)	<1	Ref.				
		1-2	0.445	0.190	–	0.699	**0.001**
		> 2	0.446	0.133	–	0.758	**0.005**
	Passive smoking	No	Ref.				
		Yes	0.359	0.053	–	0.664	**0.022**
	Coffee	No	Ref.				
		Yes	0.232	-0.028	–	0.493	0.080
	Egg (time/week)	<1	Ref.				
		1-2	-0.237	-0.530	–	0.055	0.111
		> 2	-0.010	-0.395	–	0.376	0.960
	Beer	No	Ref.				
		Yes	0.227	0.017	–	0.436	**0.034**
